# MIR205 host gene (MIR205HG) drives osteosarcoma metastasis via regulating the microRNA 2114-3p (miR-2114-3p)/twist family bHLH transcription factor 2 (TWIST2) axis

**DOI:** 10.1080/21655979.2021.1920326

**Published:** 2021-05-05

**Authors:** Xin Wang, Xiaojie Yu, Xiongwu Long, Qianqian Pu

**Affiliations:** a Department of Musculoskeletal Cancer, Hunan Cancer Hospital & the Affiliated Cancer Hospital of Xiangya School of Medicine Central South University, Changsha, PR, China; b Department of Orthopaedics, Hunan Aerospace Hospital, Changsha, China; c Department of Clinical Laboratory, Hunan Cancer Hospital & the Affiliated Cancer Hospital of Xiangya School of Medicine Central South University, Changsha, PR, China

**Keywords:** Competing endogenous RNA, metastasis, MIR205HG, osteosarcoma

## Abstract

Osteosarcoma (OS) is an aggressive malignant tumor with a high rate of lung metastasis and a lack of therapeutic targets. Although the anomalous expression of long non-coding RNA (lncRNA) has been extensively documented in human cancer, its contribution to OS metastasis remains poorly understood. In this study, we found that MIR205 host gene (MIR205HG) was significantly elevated in human OS tissues, especially in metastatic OS tissues. Stable knockdown of MIR205HG inhibited OS cell invasion and lung metastatic foci formation, but did not affect cell viability. The vast majority of MIR205HG was situated in the cytosol, and served as a competing endogenous RNA (ceRNA) that directly bound to microRNA 2114–3p (miR-2114-3p), resulting in increased twist family bHLH transcription factor 2 (TWIST2) level. Pre-clinically, high MIR205HG was linked with dismal overall and relapse-free survival. Functionally, the attenuated cell invasion caused by MIR205HG knockdown was effectively rescued by miR-2114-3p silencing or TWIST2 overexpression. Overall, our findings suggest that the previously uncharacterized regulatory axis of MIR205HG/miR-2114-3p/TWIST2 plays a critical role in promoting OS metastasis, which implies a potential therapeutic target in OS patients with metastasis.

## Graphical abstract

**Figure:**
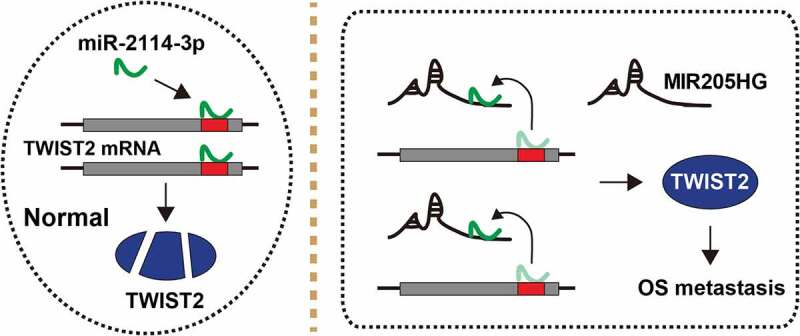

## Introduction

Osteosarcoma (OS) is one of the most common primary orthopedic malignancies [1], with a high degree of malignancy, rapid development and early pulmonary metastasis [2]. With the development of diagnostic and treatment techniques, the five-year survival rate for OS patients has increased to 60 ~ 70%, while that of OS patients with distant metastasis is still not optimistic, less than 20% [3]. Thus, it is of great impendency to clarify the mechanism responsible for OS metastasis, which will bring effective prophylaxis and treatment methods.

Long non-coding RNA (lncRNA) is a kind of RNA molecules with transcriptional length over 200 nt [4]. In mammalian genome, 4 ~ 9% of the transcripts are lncRNA, which was initially considered as the ‘noise’ of genome transcription, a by-product of RNA polymerase II transcription, without biological function [5]. However, recent studies indicate that lncRNA is widely involved in many important regulatory processes, such as chromosome silencing, genomic imprinting, chromatin modification, transcriptional activation and interference, nuclear transport and so on [6,7]. Emerging evidence shows that lncRNA is frequently abnormally expressed in human cancer [8,9], some lncRNAs activate or inactivate a series of oncogenic signals by acting as competing endogenous RNAs (ceRNAs), which carry the ‘seed sequence’ of certain miRNAs and bind miRNAs like sponges, thus preventing miRNAs from binding to their target mRNAs [10,11]. For example, lncRNA-ZFAS1 was reported as a promoter in human colorectal cancer, it could abundantly sponge miR-150-5p and increase VEGFA level, ultimately activating carcinogenic AKT/mTOR signaling pathway [12]. LncRNA ELFN1-AS1 was highly expressed in ovarian cancer cells compared to normal cells, promoted cell aggressive progression via sponging miR-497-3p and increasing CLDN4 [13]. LncRNA CAR10 was proposed as a trigger of lung adenocarcinoma cell migration and invasion, which directly bound with miR-30/miR-203 and then regulated the expression of SNAI1 and SNAI2 [14]. These studies suggest that the dysregulated ceRNA axis is critical for cancer; however, little is known about the ceRNA network involved in OS metastasis.

Research shows that MIR205HG functions as a fundamental player in various human cancer by affecting different pathways in a context-dependent manner [15,16]. In this work, the biological role of MIR205HG in OS was characterized. High MIR205HG was observed in human OS tissues, especially in metastatic OS tissues. Cell invasion and lung metastasis were evidently decreased after MIR205HG depletion. Further, the effect of MIR205HG-related ceRNA axis in OS metastasis was determined. Therefore, our study will bring new targets for the clinical treatment of metastatic OS patients.

## Materials and methods

### Tissue samples

A total of 63 pairs of adjacent normal and OS tissues (40 cases without metastasis, 23 cases with metastasis) were collected from Hunan Cancer Hospital & The Affiliated Cancer Hospital of Xiangya School of Medicine Central South University. All of them were pathologically diagnosed as OS, and did not receive any anti-tumor treatment before operation. We obtained each patient’s written informed consent and completed the follow-up.

### OS cells

HOS and MG63 cells were purchased from ATCC and routinely grown in DMEM medium supplemented with 10% FBS, and mycoplasma contamination was monitored every month. Cell transfection was conducted by using Lipofectamine 2000 (Invitrogen, CA, USA) following the supplier’s instructions. To construct stable MIR205HG-silenced cell lines, two shRNAs targeting MIR205HG were designed by RiboBio (Guangzhou, China) and inserted into pLKO.1-puro lentiviral vector, followed by co-transfection with packing plasmid (psPAX2) and envelope plasmid (pMD2G) into 293 T cells using Lipofectamine 2000. After 48 h, the supernatant virus particles were collected by centrifugation, and infected into HOS and MG63 cells, followed by selection with 1 μg/ml puromycin.

### qRT-PCR

Total RNA was isolated from OS tissues and cells using Trizol reagent (Invitrogen). After Turbo DNA-free DNase treatment (Ambion, TX, USA), reverse transcription was conducted with SuperScript® III First-Strand Synthesis system (Invitrogen) using 1 μg of RNA. Gene amplification and quantification were performed on a ViiA™ 7 Real-Time PCR System (Applied Biosystems, CA, USA). LncRNA/mRNA and miRNA expression were normalized to GAPDH and U6 levels, respectively.

### Detection of the subcellular localization of MIR205HG

The PARISTM Kit (Invitrogen) was applied to collect cytoplasmic and nuclear RNA, followed by qRT-PCR analysis. For fluorescence in situ hybridization (FISH) assay, FAM-labeled fluorescent probe targeting MIR205HG was designed by RiboBio and FISH was conducted by using Fluorescent In Situ Hybridization Kit provided by RiboBio, followed by observation of fluorescent signals under a confocal microscope.

### CCK-8 and Transwell assays

For CCK-8 assay, OS cells were seeded in 96-well plates at a density of 1,000 cells/well and allowed to grow for three consecutive days. After addition with 10 μL CCK-8 solution (Dojindo, Kumamoto, Japan) and incubation for 0.5 h at 37°C, the absorbance value of each well was recorded by using the Eon^TM^ Microplate Reader (BioTek, VT, USA). For Transwell assay, the chambers with polycarbonate membranes (Corning, CA, USA) were coated with matrigel (Corning), then, 200 μL cell suspension was added into the upper chamber, while 600 μL DMEM medium containing 10% FBS was added into the lower chamber. 24 h later, the invaded cells were fixed by methanol and stained by crystal violet.

### In vivo *lung metastasis model*

A total of 10 NOD/SCID mice (n = 5 per group) were purchased from Saike Jingda Experimental Animal Co. Ltd (Changsha, Hunan), and fed under standard pathogen-free condition. One hundred microliters cell suspension containing 1 × 10^6^ MIR205HG knockdown HOS cells were injected into mice by caudal vein. Five weeks later, 3 mg D-Luciferin were intraperitoneally injected into mice, 15 min later, the metastases were visualized and collected for H&E and immunohistochemistry (IHC) staining.

### RNA pull-down and immunoprecipitation (RIP)

For MIR205HG pulling down miRNAs, MIR205HG probe was designed and labeled with biotin (RiboBio), then added into cell lysates and incubated for 2 h at 4°C. After that, the M-280 Dynabeads Streptavidin beads (Invitrogen) were added into above RNA complexes, and incubated for 1 h at room temperature. The enriched miRNAs were washed with 50 mM Tris–HCl at pH 7.5, 150 mM NaCl, 1 mM MgCl_2_ and 0.05% NP-40 and extracted by TRIzol reagent using for qRT-PCR analysis. For miR-2114-3p pulling down MIR205HG or TWIST2 mRNA, the wild-type and mutant miR-2114-3p mimics labeled with biotin (RiboBio) were transfected into OS cells for 48 h. After incubation with M-280 Dynabeads Streptavidin beads, the enrichment of MIR205HG/TWIST2 mRNA was determined by qRT-PCR analysis. Besides, RIP assay was carried out by using Magna RIP^TM^ RNA-binding Protein Immunoprecipitation Kit (Millipore, MA, USA) following the manufacturer’s protocol.

### Luciferase reporter assay

The sequences of MIR205HG and TWIST2 3-UTR containing wild-type or mutant miR-2114-3p binding site were synthesized and inserted into psiCHECK-2 luciferase vector (Promega, WI, USA), followed by co-transfection with control or miR-2114-3p mimics into OS cells using Lipofectamine 2000. 48 h later, the luciferase activity in each well was detected.

### Immunofluorescence (IF)

OS cells were transfected with control or miR-2114-3p mimics for 48 h, then washed by PBS and fixed by 4% formaldehyde for 10 min, followed by permeabilization with 0.5% Triton X-100 for 15 min and blocking with 5% bovine serum albumin for 30 min. Afterward, 2 µg/mL anti-TWIST2 rabbit polyclonal antibody (ab247148, Abcam) was added and incubated overnight at 4°C. The next day, the goat anti-rabbit IgG labeled with Alexa Fluor® 568 (ab175471, Abcam) was added, followed by incubation for 1 h. After washing with PBST for five times, nucleus was counterstained by DAPI reagent. Immunofluorescence images were obtained by using a confocal microscope.

### Western blot

OS cells were lysed in RIPA buffer (Beyotime, Beijing, China) containing protease inhibitor cocktail tablet and phosphatase inhibitors. The lysate was subjected to SDS–PAGE and transferred onto PVDF membrane. After blocking with 5% nonfat milk powder, the membrane was incubated with anti-TWIST2 primary antibody and horseradish peroxidase-conjugated secondary antibody. Then, the band was detected by using Novex® Enhanced Chemoluminiscence Substrate Reagent Kit (Invitrogen).

### Statistical analysis

All functional experiments were repeated at least three times, and the results were shown as mean ± standard deviation. All figures were generated by Graphpad prism 7.0. The difference between two groups was determined by t test, and the correlation between MIR205HG and miR-2114-3p or TWIST2 in OS tissues was assessed by Spearman correlation coefficient. Kaplan-Meier plotter was used to test the effect of MIR205HG expression on OS patient’s overall and relapse survival. *P*< 0.05 was considered to be statistically significant.

## Results

This study aimed to describe the role of MIR205HG in OS. Through performing a cohort of functional assays, including CCK-8, Transwell and animal model, it was found that MIR205HG promoted OS cell metastasis but had no effect on cell growth. Further studies suggested that MIR205HG positively correlated with TWIST2, while negatively correlated with miR-2114-3p in OS tissues, which was due to the sponge effect of MIR205HG on miR-2114-3p. Our data highlight the importance of MIR205HG in OS metastasis, and dysregulation of MIR205HG/miR-2114-3p/TWIST2 ceRNA axis may be critical for OS cell invasion.

### MIR205HG is significantly upregulated in OS tissues

**Figure 1.**
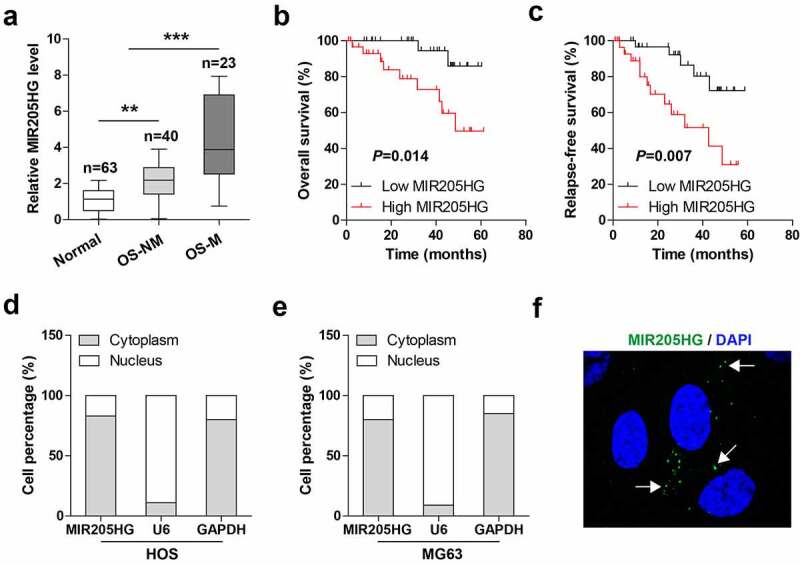
High MIR205HG is identified in human OS tissues. (a). qRT-PCR analysis of MIR205HG level in normal and OS tissues; OS-NM: non-metastatic OS tissues; OS-M: metastatic OS tissues. (b, c). The survival curve of OS patients based on median MIR205HG level. (d–f). qRT-PCR analysis and FISH assay testing the subcellular localization of MIR205HG in OS cells. **P< 0.01, ***P< 0.001

The levels of MIR205HG in OS and normal tissues were tested by qRT-PCR, MIR205HG was shown to be evidently elevated in OS as compared to normal tissues (Figure 1(a)), of note, higher MIR205HG expression was observed in metastatic OS tissues (Figure 1(a)). The survival curve showed that high MIR205HG was correlated with poor overall and relapse-free survival (Figure 1(b,c)). Next, the subcellular localization of MIR205HG in OS cells was determined, and the results of Figure 1(d and e) showed that MIR205HG was a cytoplasmic lncRNA in both HOS and MG63 cells, which was also verified by FISH assay (Figure 1(f)). These data suggest that MIR205HG is mainly localized in the cytoplasm, and may be linked to OS metastasis.

### Stable silencing of MIR205HG inhibits OS cell invasion and metastasis

**Figure 2.**
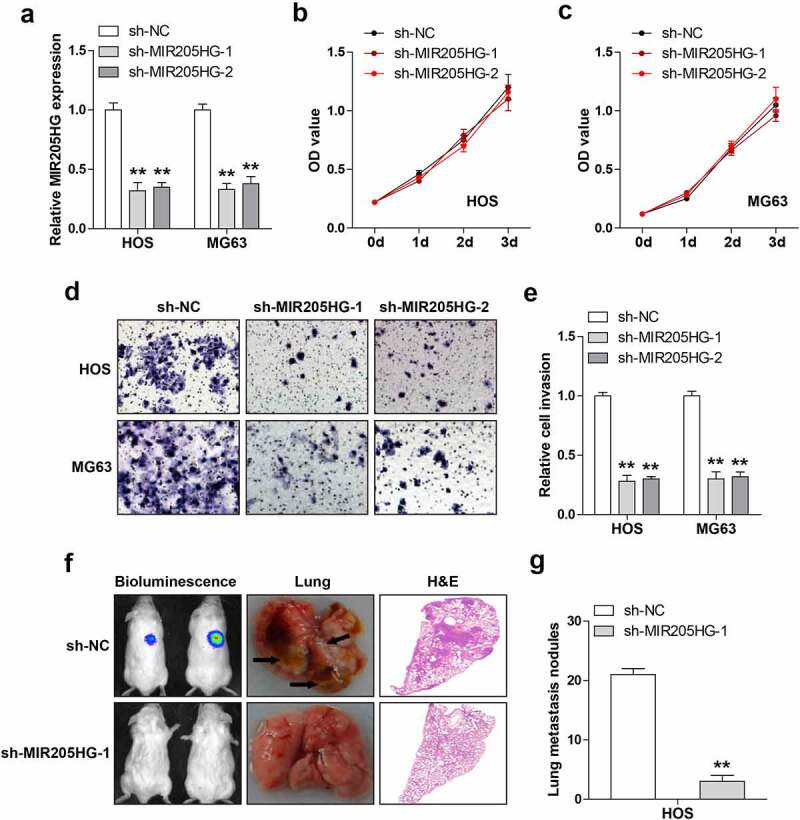
MIR205HG knockdown represses cell invasion and metastasis. (a). qRT-PCR analysis of MIR205HG levels in OS cells after infection with lentiviral vector. (b, c). CCK-8 assay testing cell viability after MIR205HG silencing. (d, e). Transwell assay testing cell invasion after MIR205HG silencing. (f, g). Lung metastasis assay testing the effect of MIR205HG on OS cell metastasis *in vivo*. **P< 0.01

Then, the lentiviral vector was infected into OS cells to obtain stable MIR205HG knockdown cell lines (Figure 2(a)). As shown in Figure 2(b and c), MIR205HG knockdown did not affect cell viability in both HOS and MG63 cells. However, based on Figure 2(d and e), cell invasion was significantly attenuated after depletion of MIR205HG. To test whether MIR205HG also functioned *in vivo*, MIR205HG-silenced HOS cells were injected into NOD/SCID mice and routinely fed them for five weeks. The results showed that the number of metastatic foci in MIR205HG knockdown group was significantly less than that in control group (Figure 2(f,g)). These data indicate that MIR205HG is a pro-metastasis lncRNA in OS.

### MIR205HG functions as a ceRNA sponging miR-2114-3p in OS cells

**Figure 3.**
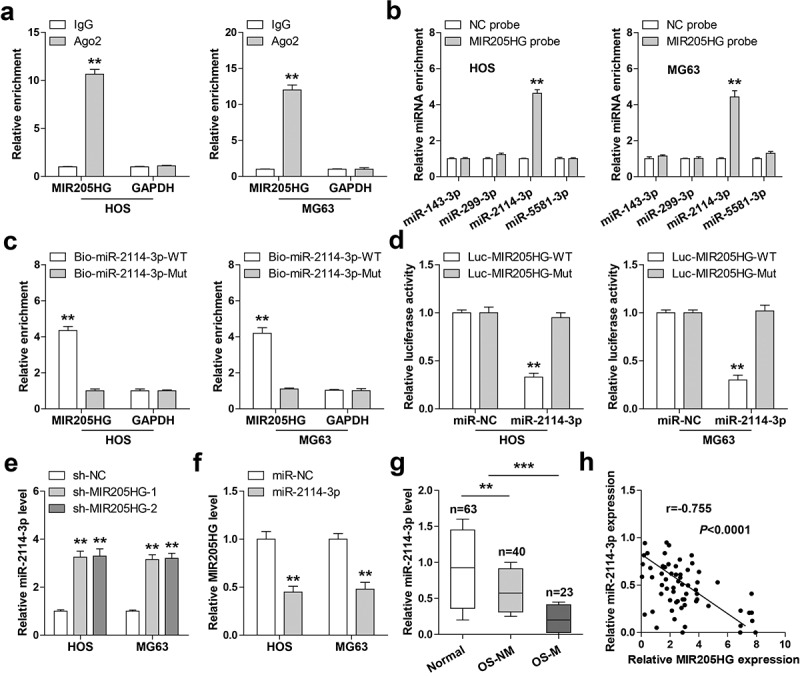
MIR205HG sponges miR-2114-3p. (a). RIP assay in OS cells with anti-Ago2 antibody, followed by qRT-PCR analysis of MIR205HG and GAPDH enrichment. (b,c). RNA pull-down assay using biotin-labeled MIR205HG/miR-2114-3p probe, followed by qRT-PCR analysis of relative enrichment. (d). Luciferase reporter assay detecting the binding of miR-2114-3p on MIR205HG. (e, f). qRT-PCR analysis of miR-2114-3p/MIR205HG levels in MIR205HG-silenced/miR-2114-3p-overexpressed OS cells. (g). qRT-PCR analysis of miR-2114-3p in normal and OS tissues. H. The correlation between MIR205HG and miR-2114-3p in OS tissues. **P< 0.01, ***P< 0.001

In light of the cytosolic location of MIR205HG, it was conjectured that MIR205HG might be a ceRNA. By performing Ago2-RIP assay, we found that MIR205HG, not GAPDH, was abundantly immunoprecipitated by Ago2 protein (Figure 3(a)), a core component of RNA-induced silencing complex (RISC) that is essential for miRNA function. Then, by analyzing the Starbase 3.0 containing the CLIP-seq data of Ago2 (http://www.sysu.edu.cn), it was found that four miRNAs might interact with MIR205HG. RNA pull-down assay showed that only miR-2114-3p was significantly enriched by MIR205HG probe in both HOS and MG63 cells (Figure 3(b)). Consistently, miR-2114-3p probe could also pull down endogenous MIR205HG, but this effect disappeared by mutated probe (Figure 3(c)). Furthermore, overexpression of miR-2114-3p substantially reduced the luciferase activity of wild-type MIR205HG vector, while had no effect on the mutant one (Figure 3(d)). Knockdown of MIR205HG evidently increased miR-2114-3p levels (Figure 3(e)), and miR-2114-3p overexpression decreased MIR205HG levels in both HOS and MG63 cells (Figure 3(f)). Besides, MIR205HG was dramatically downregulated in OS tissues, especially in metastatic tissues (Figure 3(g)). Importantly, a strong negative correlation between MIR205HG and miR-2114-3p was found in OS tissues (r = −0.755) (Figure 3(h)). These data suggest that MIR205HG serves as a sponge of miR-2114-3p and inhibit miR-2114-3p in OS cells.

### MIR205HG sponges miR-2114-3p to elevate TWIST2 in OS cells

**Figure 4.**
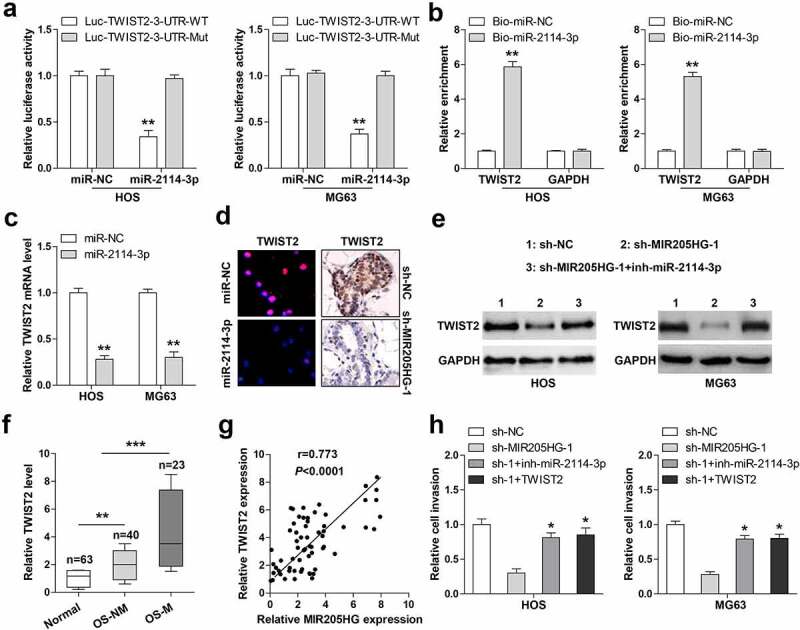
MIR205HG elevates TWIST2 via miR-2114-3p. (a). Luciferase reporter assay detecting the binding of miR-2114-3p on TWIST2 3`-UTR. (b). RNA pull-down assay using biotin-labeled miR-2114-3p probe, followed by qRT-PCR analysis of relative enrichment. (c). qRT-PCR analysis of TWIST2 mRNA level in miR-2114-3p-overexpressed OS cells. (d). IF and IHC assays testing TWIST2 protein level in miR-2114-3p-overexpressed OS cells and metastatic lung tissues. (e). Western blot assay testing TWIST2 protein level in stable MIR205HG-silenced OS cells transfected with miR-2114-3p inhibitors. (f). qRT-PCR analysis of TWIST2 mRNA level in normal and OS tissues. (g). The correlation between MIR205HG and TWIST2 in OS tissues. (h). Transwell assay testing cell invasion in stable MIR205HG-silenced OS cells transfected with miR-2114-3p inhibitors or TWIST2-overexpressed vector. *P< 0.05, **P< 0.01, ***P< 0.001

Through analyzing the microCLIP online algorithm (http://carolina.imis.athena-innovation.gr/) containing AGO2-PAR-CLIP sequencing data, it was found that TWIST2, a key driver of cancer metastasis, might be the target of miR-2114-3p. Luciferase reporter assay results showed that miR-2114-3p overexpression significantly weakened the luciferase activity of wild-type TWIST2 3`-UTR vector, but this effect was blocked by mutant vector (Figure 4(a)). Likewise, miR-2114-3p probe could pull down TWIST2 3`-UTR in both HOS and MG63 cells (Figure 4(b)). TWIST2 mRNA (Figure 4(c)) and protein (Figure 4(d)) levels were notably reduced after miR-2114-3p overexpression. Importantly, knockdown of MIR205HG also resulted in a significant decrease in TWIST2 expression in lung metastasis models (Figure 4(d)) and cells (Figure 4(e)), whereas silencing of miR-2114-3p effectively counteracted this phenomenon (Figure 4(e)). High TWIST2 was observed in OS tissues, especially in metastatic tissues (Figure 4(f)). And OS tissues with high MIR205HG expression presented high TWIST2 level (r = 0.773) (Figure 4(g)). Importantly, miR-2114-3p depletion or TWIST2 reintroduction partially rescued the declined cell invasion caused by MIR205HG knockdown (Figure 4(h)). These data imply that MIR205HG indirectly increased TWIST2 levels in OS cells via sponging and inhibiting miR-2114-3p activity.

## Discussion

**Figure 5.**
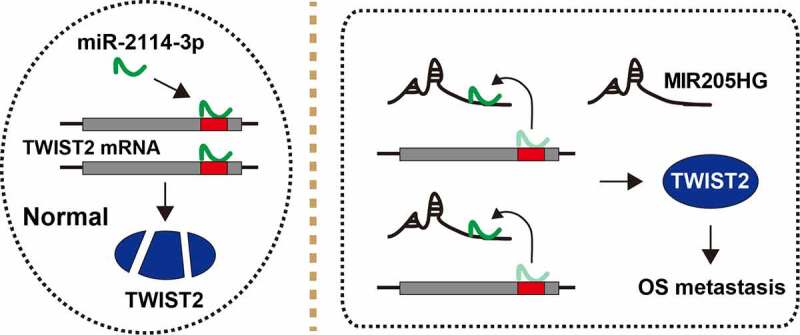
The schematic diagram of the action of MIR205HG in OS. In normal cells, miR-2114-3p binds to TWIST2 mRNA 3`-UTR, resulting in decreased TWIST2 level; In OS cells, MIR205HG sponges miR-2114-3p, antagonizing the inhibitory effect of miR-2114-3p on TWIST2, thereby increasing TWIST2 expression and subsequently promoting OS metastasis

LncRNA is crucial for cancer initiation, development and progression [17]. Likewise, some lncRNAs have been found to be key regulators in OS progression, such as MRPL23-AS1 [18] and XIST [19]. In the present study, a lncRNA MIR205HG was identified to link to OS invasion and metastasis. MIR205HG was significantly increased in human OS tissues, and predicted poor prognosis. Depletion of MIR205HG evidently attenuated cell invasion, and *in vivo* experiment further showed that MIR205HG knockdown reduced OS cell lung metastasis. In terms of the mechanism, MIR205HG was able to directly bind to miR-2114-3p, relieving the suppressive effect of miR-2114-3p on TWIST2 mRNA, leading to elevated TWIST2 expression and OS distant dissemination (Figure 5). In addition, MIR205HG was conversely correlated with miR-2114-3p, and positively correlated with TWIST2 in OS tissues, implying that this MIR205HG/miR-2114-3p/TWIST2 ceRNA axis does exist and is deregulated in OS. Therefore, our findings advance the understanding of the importance of lncRNA in cancer biology, and also provide experimental evidence for lncRNA as a model of ceRNA.

The function of lncRNA depends on its subcellular localization, nuclear lncRNA mainly regulates histone modification and chromatin status by binding to protein or DNA, affecting gene expression at the transcriptional level; cytoplasmic lncRNA can interact with protein, miRNA, DNA or RNA, regulating gene location, stability, or even protein translation [20,21]. Among them, the model of lncRNA sponging miRNA is widely proven [22]. miRNA is a single-stranded RNA molecule encoded by endogenous gene with a length of about 22 nucleotides, can bind to the 3ʹ-UTR region of target genes by base complementary matching, leading to reducing the stability of target genes or inhibiting their translation, thus playing a role in post-transcriptional regulation of gene expression [23,24]. Mature miRNA is transferred from nucleus to cytoplasm and loaded into RISC via Ago2 protein to exert its gene silencing function [25]. Thus, the prerequisite of lncRNA as a ceRNA is that it locates in the cytoplasm and interacts with RISC. Herein, MIR205HG was shown to be a cytosolic lncRNA in OS cells, and RIP assay showed that MIR205HG abundantly interacted with Ago2 protein, indicating that MIR205HG may function via sponging miRNAs. As expected, a series of subsequent bioanalysis and assays showed that miR-2114-3p was the target of MIR205HG, MIR205HG inhibited the activity of miR-2114-3p to increase the level of TWIST2. TWIST2 is a well-known metastatic trigger in various human cancer types, which is frequently overexpressed in cancer tissues and correlated with poor prognosis [26]. Here, TWIST2 was confirmed to be significantly increased in OS tissues, especially in metastatic tissues, and its expression was inhibited by miR-2114-3p overexpression or MIR205HG knockdown. These data suggest that MIR205HG is a ceRNA in OS that sponges miR-2114-3p and upregulates TWIST2 expression.

It is noteworthy that lncRNA expression and function vary in a cell-, tissue- and developmental-stage specific pattern [27]. For example, the star lncRNA HOTAIR has been reported to be a driver of hepatocarcinogenesis via miR-218 [28]; however, it respectively bound to androgen receptor [29], miR-149-5p [30], miR-206 [31], miR-601 [32], and miR-1277-5p [33] in renal cell carcinoma, non-small cell lung cancer, ovarian cancer and breast cancer, exerting its pro-tumor role. Likewise, studies have shown that MIR205HG functioned as an oncogene in esophageal cancer, cervical cancer and head & neck squamous cell carcinoma by interacting with miR-214 [16], SRSF1 [34] and miR-590-3p [35], respectively. Here, MIR205HG was also a carcinogenic lncRNA in OS, it promoted OS metastasis, but did not affect cell viability, hinting the different functions of lncRNA relying on different contexts. Further study is needed to explore the expression, clinical implication and biological function of MIR205HG in other malignant tumors.

## Conclusion

In sum, our study deciphers the previously uncharacterized pro-metastasis function of MIR205HG in OS, targeting of the deregulated MIR205HG/miR-2114-3p/TWIST2 ceRNA axis may be utilized as a promising therapeutic strategy for OS patients with metastasis.

## Supplementary Material

Supplemental Material

## Data Availability

The datasets used and/or analysed for the current study are available from the corresponding author upon reasonable request.

## Highlights

1. MIR205HG was notably upregulated in OS tissues, especially in metastatic tissues.
2. Knockdown of MIR205HG weakened OS cell invasion and metastasis.
3. MIR205HG sponged miR-2114-3p in OS cells.
4. MIR205HG regulated the miR-2114-3p/TWIST2 signaling in OS cells.
