# Supplementary figures and images for: MIR205 host gene (MIR205HG) drives osteosarcoma metastasis via regulating the microRNA 2114-3p (miR-2114-3p)/twist family bHLH transcription factor 2 (TWIST2) axis
Source: Bioengineered. 2021 May 5;12(1):1576–86. doi: 10.1080/21655979.2021.1920326 (PMC8806225; doi:10.1080/21655979.2021.1920326)

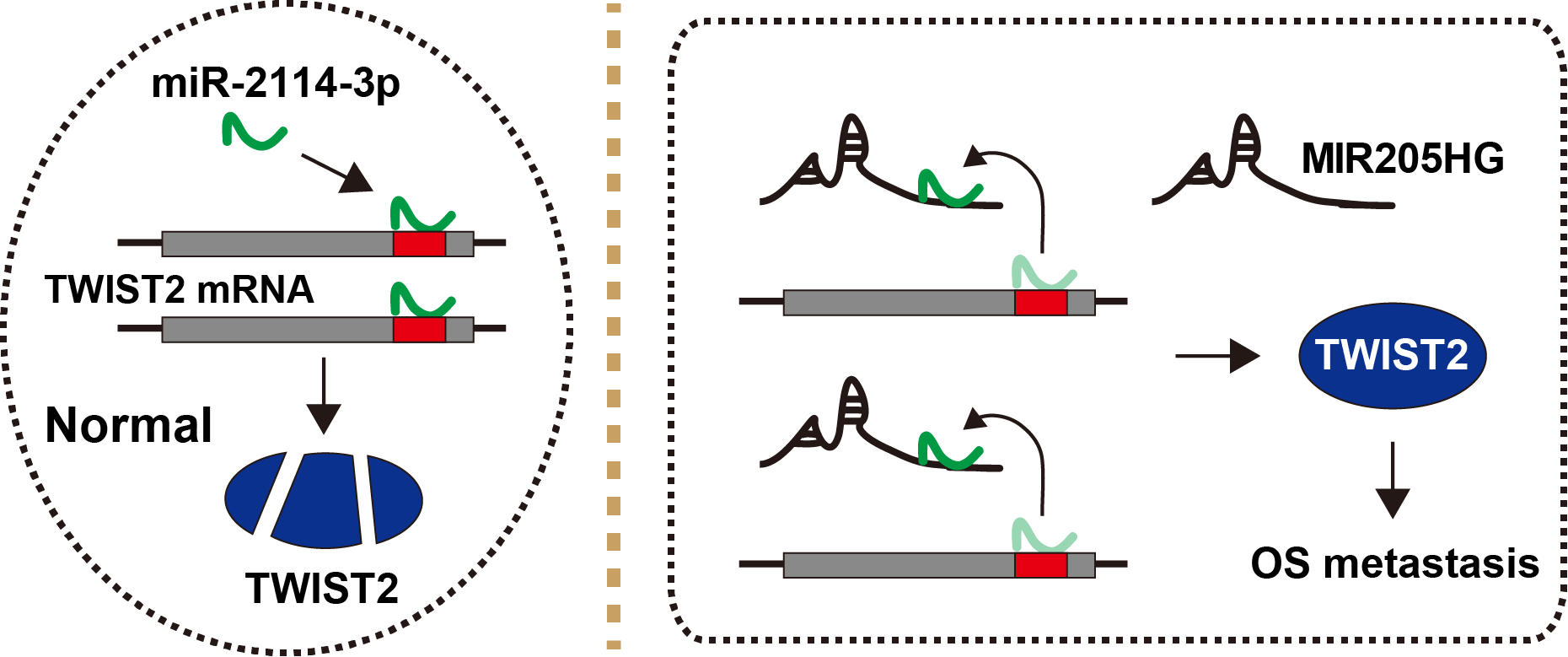

Supplement: Supplemental Material [file KBIE_A_1920326_SM4446.tif]
